# Oxyresveratrol from Mulberry as a dihydrate

**DOI:** 10.1107/S1600536812014018

**Published:** 2012-04-06

**Authors:** Hui Deng, Xixin He, Yujuan Xu, Xiaopeng Hu

**Affiliations:** a School of Pharmaceutical Science, Sun Yat-sen University, Guangzhou 510006, People’s Republic of China; b College of Chinese Materia Medica, Guangzhou University of Chinese Medicine, Guangzhou 510006, People’s Republic of China

## Abstract

The title compound {systematic name: 4-[(*E*)-2-(3,5-dihy­droxy­phen­yl)ethen­yl]benzene-1,3-diol dihydrate}, C_14_H_12_O_4_·2H_2_O, a derivative of resveratrol, was isolated from mulberry. The linking C=C double bond has a *trans* conformation and allows the formation of a conjugated system throughout the mol­ecule. The dihedral angle between the benzene rings is 9.39 (9)°. In the crystal, mol­ecules are connected into a three-dimensional architecture through O—H⋯O hydrogen bonds between hy­droxy groups of oxyresveratrol and solvent water mol­ecules.

## Related literature

For medicinal properties and the biological activity of oxyresveratrol, see: Mongolsuk *et al.* (1957 ▶); Charoenlarp *et al.* (1981 ▶, 1989 ▶); Zheng *et al.* (2010 ▶, 2011 ▶); Kim *et al.* (2002 ▶, 2004 ▶); Shin *et al.* (1998 ▶); Lipipun *et al.* (2011 ▶); Galindo *et al.* (2011 ▶); Sasivimolphan *et al.* (2009 ▶); Chuanasa *et al.* (2008 ▶); Likhitwitayawuid (2008 ▶); Likhitwitayawuid *et al.* (2005 ▶, 2006 ▶); Liu *et al.* (2009 ▶); Breuer *et al.* (2006 ▶); Chung *et al.* (2003 ▶); Chao *et al.* (2008 ▶); Ban *et al.* (2006 ▶, 2008 ▶); Breuer *et al.* (2006 ▶); Andrabi *et al.* (2004 ▶). For related structures, see: Piao *et al.* (2009 ▶); Qiu *et al.*(1996 ▶); Hano *et al.* (1986 ▶).
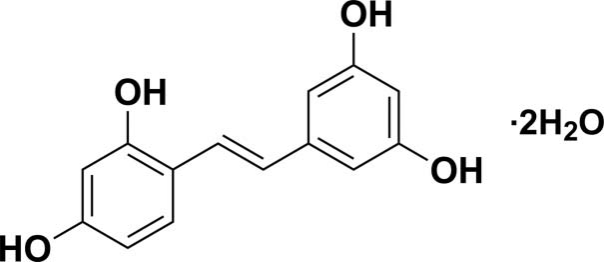


## Experimental

### 

#### Crystal data

C_14_H_12_O_4_·2H_2_O
*M* *_r_* = 280.27
Triclinic, 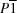

*a* = 6.6523 (5) Å
*b* = 9.2005 (9) Å
*c* = 11.5294 (8) Å
α = 72.533 (7)°
β = 78.686 (6)°
γ = 79.651 (7)°
*V* = 654.51 (9) Å^3^
*Z* = 2
Cu *K*α radiation
μ = 0.95 mm^−1^
*T* = 293 K
0.40 × 0.30 × 0.20 mm

#### Data collection

Agilent Xcalibur Onyx Nova diffractometer
Absorption correction: multi-scan (*CrysAlis PRO*; Agilent, 2010 ▶) *T*_min_ = 0.704, *T*_max_ = 0.834
4145 measured reflections
2297 independent reflections
2002 reflections with *I* > 2σ(*I*)
*R*_int_ = 0.022

#### Refinement

*R*[*F* ^2^ > 2σ(*F* ^2^)] = 0.042
*wR*(*F* ^2^) = 0.141
*S* = 1.13
2297 reflections
197 parameters
5 restraints
H atoms treated by a mixture of independent and constrained refinement
Δρ_max_ = 0.50 e Å^−3^
Δρ_min_ = −0.44 e Å^−3^

### 

Data collection: *CrysAlis PRO* (Agilent, 2010 ▶); cell refinement: *CrysAlis PRO*; data reduction: *CrysAlis PRO*; program(s) used to solve structure: *SHELXTL* (Sheldrick, 2008 ▶); program(s) used to refine structure: *SHELXTL*; molecular graphics: *SHELXTL*; software used to prepare material for publication: *publCIF* (Westrip, 2010 ▶).

## Supplementary Material

Crystal structure: contains datablock(s) I, global. DOI: 10.1107/S1600536812014018/tk5080sup1.cif

Supplementary material file. DOI: 10.1107/S1600536812014018/tk5080Isup2.mol

Structure factors: contains datablock(s) I. DOI: 10.1107/S1600536812014018/tk5080Isup3.hkl

Supplementary material file. DOI: 10.1107/S1600536812014018/tk5080Isup4.cml

Additional supplementary materials:  crystallographic information; 3D view; checkCIF report

## Figures and Tables

**Table 1:** Hydrogen-bond geometry (Å, °)

*D*—H⋯*A*	*D*—H	H⋯*A*	*D*⋯*A*	*D*—H⋯*A*
O1—H1⋯O4^i^	0.84	1.90	2.7329 (18)	173
O2—H2⋯O6^ii^	0.84	1.89	2.720 (2)	171
O3—H3*A*⋯O6^iii^	0.84	1.97	2.8045 (19)	169
O4—H4⋯O5^iv^	0.84	1.89	2.7259 (19)	170
O5—H5*A*⋯O1^i^	0.89 (2)	1.90 (2)	2.7879 (19)	172 (2)
O5—H5*B*⋯O2^v^	0.89 (2)	1.88 (2)	2.7449 (19)	161 (2)
O6—H6*B*⋯O2^ii^	0.90 (3)	1.95 (3)	2.720 (2)	143 (4)
O6—H6*C*⋯O6^vi^	0.90 (4)	1.87 (4)	2.7583 (19)	172 (5)

Symmetry codes: (i) 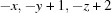; (ii) 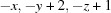; (iii) 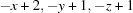; (iv) 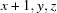; (v) 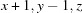; (vi) 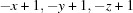.

**Fig. 1.**
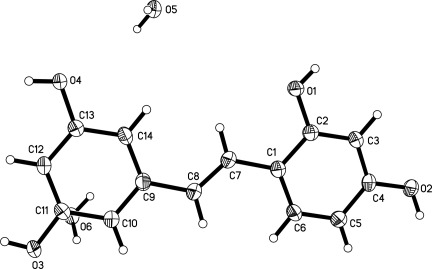
A view of the molecular structure of compound (I). The displacement ellipsoids are at the 50% probability level and H atoms are shown as small spheres of arbitrary radii.

## supplementary crystallographic information

### Comment

Oxyresveratrol was firstly isolated from the heartwood of *Artocarpus lakoocha* Roxb as a abundant *trans*-tetrahydroxystilbene (Mongolsuk *et al.*, 1957). Oxyresveratrol is responsible for the anthelmintic activity of the traditional Thailand anthelmintic drug "Puag-Haad" prepared from *A. lakoocha* (Charoenlarp *et al.*, 1989; Charoenlarp *et al.*, 1981). Recent investigations have revealed several interesting bioactivities of oxyresveratrol, such as tyrosinase inhibitory activity (Zheng *et al.*, 2011; Zheng *et al.*, 2010; Kim *et al.*, 2004; Kim *et al.*, 2002; Shin *et al.*, 1998), *in vitro* anti-viral activity (Lipipun *et al.*, 2011; Galindo *et al.*, 2011; Sasivimolphan *et al.*, 2009; Chuanasa *et al.*, 2008; Likhitwitayawuid *et al.*, 2006; Likhitwitayawuid *et al.*, 2005), strong anti-oxidative and anti-inflammatory (Liu *et al.*, 2009; Breuer *et al.*, 2006; Chung *et al.*, 2003), and neuroprotective properties (Chao *et al.*, 2008; Ban *et al.*, 2008; Ban *et al.*, 2006; Breuer *et al.*, 2006; Andrabi *et al.*, 2004). These medicinal properties indicate several areas of therapeutic potential for oxyresveratrol and the compound has been recommended as a drug candidate for the treatment of neurodegenerative disorders (Breuer *et al.*, 2006; Andrabi *et al.*, 2004) and a skin-whitening agent in cosmetic preparations (Likhitwitayawuid, 2008). Mulberry (Morus alba *L*.) is a medicinal plant in east Asia. Its branch, leaf, ripe fruit and root bark are well known traditional Chinese drugs and contain a large amounts of *trans*-hydroxystilbenes such as mulberroside A, phaponticin, phapontigenin, resveratrol, pterostilbene, piceatannol, piceid, astringin, kuwanon Y, kuwanon *Z*, oxyresveratrol and its derivatives (Qiu *et al.*, 1996; Hano *et al.*, 1986; Piao *et al.*, 2009). In this paper, we isolated oxyresveratrol from mulberry (*Morus alba L*.) and report the structure of oxyresveratrol dihydrate.

In the title compound (Fig. 1), the benzene rings form a dihedral angle of 9.39 (9)°. The presence of the *trans* C═C double bond allows the formation of a conjugated system, strongly stabilized through π-electron delocalization. The *trans*-double bond is the same as found in similar structures (Piao *et al.*, 2009; Qiu *et al.*, 1996; Hano *et al.*, 1986). The molecules of the oxyresveratrol are connected into a three-dimensional architecture through O—H—O hydrogen bonds formed between its hydroxyl group and the solvent water molecules (Fig. 2 and Table 1).

### Experimental

The dried root bark of Morus alba *L*. (1 kg) was powdered and extracted with 95% ethanol at room temperature for 48 h. After removal of the solvent under reduced pressure, a brown extract was suspended with water, and sequentially partitioned with petroleum ether, acetyl acetate and n-butanol. The acetyl acetate extract (9 g) was subjected to column chromatography on silica gel (200–300 mesh) with increasing concentrations of ethyl acetate in petroleum ether. Oxyresveratrol was afforded from the fraction (petroleum ether-ethyl acetate 6/4, *v*/*v*). The title compound was colourless to light-yellow crystal with *M*.pt: 474–476 K,. Crystals suitable for X-ray analysis were obtained by slow evaporation from its chloroform–methanol (4/1, *v*/*v*) solution. ^1^H NMR (400 MHz, MeOD) δ 7.33 (d, J = 9.2 Hz, 1H), 7.27 (d, J = 16.5 Hz, 1H), 6.82 (d, J = 16.4 Hz,1*H*), 6.46 (d, J = 2.1 Hz, 2H), 6.34 – 6.32 (m, 1H), 6.31 (s, 1H), 6.15 (t, J = 2.2 Hz, 1H); O—H not observed.

### Refinement

The hydroxy- and C-bound H-atoms were placed in calculated positions [O—H = 0.84 Å, *U*_iso_(H) = 1.5*U*_eq_(O); C—H = 0.95 Å, *U*_iso_(H) = 1.2*U*_eq_(C)] and were included in the refinement in the riding model approximation. The water-H atoms were refined with O—H = 0.90±0.01 Å, and with *U*_iso_(H) = 1.5*U*_eq_(O). The O6-water molecule was found to be disordered over three positions, one with full weight, the others with 0.5 site occupancy factors.

### Crystal data

**Table:**

C_14_H_12_O_4_·2H_2_O	*Z* = 2
*M**_r_* = 280.27	*F*(000) = 296
Triclinic, *P*1	*D*_x_ = 1.422 Mg m^−^^3^
Hall symbol: -P 1	Cu *K*α radiation, λ = 1.54178 Å
*a* = 6.6523 (5) Å	Cell parameters from 2627 reflections
*b* = 9.2005 (9) Å	θ = 4.1–71.3°
*c* = 11.5294 (8) Å	µ = 0.95 mm^−^^1^
α = 72.533 (7)°	*T* = 293 K
β = 78.686 (6)°	Block, colourless
γ = 79.651 (7)°	0.40 × 0.30 × 0.20 mm
*V* = 654.51 (9) Å^3^	

### Data collection

**Table:**

Agilent Xcalibur Onyx Nova diffractometer	2297 independent reflections
Radiation source: Nova (Cu) X-ray Source	2002 reflections with *I* > 2σ(*I*)
Mirror monochromator	*R*_int_ = 0.022
Detector resolution: 8.2417 pixels mm^-1^	θ_max_ = 66.6°, θ_min_ = 4.1°
ω scans	*h* = −7→8
Absorption correction: multi-scan (*CrysAlis PRO*; Agilent, 2010)	*k* = −10→8
*T*_min_ = 0.704, *T*_max_ = 0.834	*l* = −13→13
4145 measured reflections	

### Refinement

**Table:**

Refinement on *F*^2^	Primary atom site location: structure-invariant direct methods
Least-squares matrix: full	Secondary atom site location: difference Fourier map
*R*[*F*^2^ > 2σ(*F*^2^)] = 0.042	Hydrogen site location: inferred from neighbouring sites
*wR*(*F*^2^) = 0.141	H atoms treated by a mixture of independent and constrained refinement
*S* = 1.13	*w* = 1/[σ^2^(*F*_o_^2^) + (0.081*P*)^2^ + 0.176*P*] where *P* = (*F*_o_^2^ + 2*F*_c_^2^)/3
2297 reflections	(Δ/σ)_max_ < 0.001
197 parameters	Δρ_max_ = 0.50 e Å^−^^3^
5 restraints	Δρ_min_ = −0.44 e Å^−^^3^

### Special details

**Table:**

Geometry. All e.s.d.'s (except the e.s.d. in the dihedral angle between two l.s. planes) are estimated using the full covariance matrix. The cell e.s.d.'s are taken into account individually in the estimation of e.s.d.'s in distances, angles and torsion angles; correlations between e.s.d.'s in cell parameters are only used when they are defined by crystal symmetry. An approximate (isotropic) treatment of cell e.s.d.'s is used for estimating e.s.d.'s involving l.s. planes.
Refinement. Refinement of *F*^2^ against ALL reflections. The weighted *R*-factor *wR* and goodness of fit *S* are based on *F*^2^, conventional *R*-factors *R* are based on *F*, with *F* set to zero for negative *F*^2^. The threshold expression of *F*^2^ > σ(*F*^2^) is used only for calculating *R*-factors(gt) *etc*. and is not relevant to the choice of reflections for refinement. *R*-factors based on *F*^2^ are statistically about twice as large as those based on *F*, and *R*- factors based on ALL data will be even larger.

### Fractional atomic coordinates and isotropic or equivalent isotropic displacement parameters (Å^2^)

**Table:**

	*x*	*y*	*z*	*U*_iso_*/*U*_eq_	Occ. (<1)
C1	−0.1126 (3)	0.9054 (2)	0.76812 (16)	0.0253 (4)	
C2	−0.2896 (3)	0.8772 (2)	0.85658 (16)	0.0251 (4)	
C3	−0.4773 (3)	0.9699 (2)	0.84350 (16)	0.0260 (4)	
H3	−0.5945	0.9490	0.9051	0.031*	
C4	−0.4925 (3)	1.0933 (2)	0.73988 (17)	0.0270 (4)	
C5	−0.3191 (3)	1.1277 (2)	0.65217 (18)	0.0304 (4)	
H5	−0.3286	1.2143	0.5825	0.036*	
C6	−0.1329 (3)	1.0347 (2)	0.66730 (17)	0.0291 (4)	
H6	−0.0149	1.0594	0.6073	0.035*	
C7	0.0785 (3)	0.7976 (2)	0.78124 (16)	0.0265 (4)	
H7	0.0724	0.7073	0.8482	0.032*	
C8	0.2599 (3)	0.8143 (2)	0.70802 (17)	0.0266 (4)	
H8	0.2682	0.9072	0.6438	0.032*	
C9	0.4478 (3)	0.7025 (2)	0.71754 (16)	0.0241 (4)	
C10	0.6184 (3)	0.7319 (2)	0.62552 (16)	0.0258 (4)	
H10	0.6122	0.8242	0.5605	0.031*	
C11	0.7979 (3)	0.6264 (2)	0.62855 (16)	0.0257 (4)	
C12	0.8084 (3)	0.4895 (2)	0.72244 (17)	0.0273 (4)	
H12	0.9292	0.4163	0.7238	0.033*	
C13	0.6385 (3)	0.4623 (2)	0.81404 (16)	0.0260 (4)	
C14	0.4594 (3)	0.5662 (2)	0.81327 (16)	0.0257 (4)	
H14	0.3454	0.5451	0.8771	0.031*	
O1	−0.27079 (18)	0.75380 (14)	0.95877 (12)	0.0298 (3)	
H1	−0.3888	0.7321	0.9944	0.045*	
O2	−0.68101 (19)	1.17941 (15)	0.72308 (13)	0.0352 (4)	
H2	−0.6634	1.2691	0.6802	0.053*	
O3	0.95691 (19)	0.66150 (15)	0.53537 (12)	0.0316 (3)	
H3A	1.0617	0.5971	0.5509	0.047*	
O4	0.64072 (19)	0.32934 (15)	0.90904 (12)	0.0330 (4)	
H4	0.7589	0.2786	0.9044	0.049*	
O5	0.01297 (19)	0.14910 (15)	0.92354 (12)	0.0318 (3)	
H5A	0.085 (3)	0.187 (3)	0.963 (2)	0.048*	
H5B	0.095 (3)	0.148 (3)	0.8524 (14)	0.048*	
O6	0.67051 (19)	0.52209 (15)	0.40983 (12)	0.0300 (3)	
H6A	0.637 (4)	0.490 (3)	0.3506 (17)	0.045*	
H6B	0.701 (7)	0.618 (2)	0.394 (4)	0.045*	0.50
H6C	0.565 (6)	0.513 (6)	0.472 (3)	0.045*	0.50

### Atomic displacement parameters (Å^2^)

**Table:**

	*U*^11^	*U*^22^	*U*^33^	*U*^12^	*U*^13^	*U*^23^
C1	0.0192 (9)	0.0256 (9)	0.0278 (9)	−0.0011 (7)	−0.0025 (7)	−0.0043 (7)
C2	0.0202 (8)	0.0243 (9)	0.0270 (9)	−0.0018 (6)	−0.0028 (7)	−0.0025 (7)
C3	0.0182 (8)	0.0268 (9)	0.0285 (9)	−0.0011 (7)	0.0012 (7)	−0.0051 (7)
C4	0.0187 (8)	0.0261 (9)	0.0322 (9)	0.0029 (7)	−0.0031 (7)	−0.0057 (7)
C5	0.0249 (9)	0.0280 (9)	0.0300 (9)	0.0003 (7)	−0.0011 (7)	0.0003 (7)
C6	0.0190 (8)	0.0307 (10)	0.0301 (9)	−0.0006 (7)	0.0017 (7)	−0.0023 (8)
C7	0.0220 (9)	0.0234 (9)	0.0295 (9)	0.0010 (7)	−0.0047 (7)	−0.0023 (7)
C8	0.0217 (9)	0.0224 (9)	0.0314 (9)	0.0006 (7)	−0.0037 (7)	−0.0034 (7)
C9	0.0204 (8)	0.0234 (9)	0.0280 (9)	−0.0009 (6)	−0.0029 (7)	−0.0080 (7)
C10	0.0211 (9)	0.0239 (9)	0.0290 (9)	−0.0010 (7)	−0.0025 (7)	−0.0042 (7)
C11	0.0195 (8)	0.0280 (9)	0.0273 (9)	−0.0042 (7)	−0.0002 (7)	−0.0056 (7)
C12	0.0182 (8)	0.0271 (9)	0.0318 (10)	0.0018 (7)	−0.0029 (7)	−0.0043 (7)
C13	0.0205 (8)	0.0266 (9)	0.0269 (9)	−0.0025 (7)	−0.0021 (7)	−0.0029 (7)
C14	0.0175 (8)	0.0303 (9)	0.0261 (9)	−0.0009 (7)	0.0003 (7)	−0.0066 (7)
O1	0.0184 (6)	0.0289 (7)	0.0309 (7)	−0.0003 (5)	−0.0007 (5)	0.0049 (5)
O2	0.0210 (7)	0.0280 (7)	0.0427 (8)	0.0060 (5)	−0.0017 (6)	0.0036 (6)
O3	0.0180 (6)	0.0314 (7)	0.0341 (7)	0.0013 (5)	0.0036 (5)	0.0003 (5)
O4	0.0189 (6)	0.0316 (7)	0.0333 (7)	0.0035 (5)	0.0022 (5)	0.0059 (6)
O5	0.0238 (7)	0.0339 (7)	0.0329 (7)	0.0019 (5)	−0.0046 (5)	−0.0050 (6)
O6	0.0239 (7)	0.0299 (7)	0.0343 (8)	−0.0026 (5)	−0.0023 (5)	−0.0078 (6)

### Geometric parameters (Å, º)

**Table:**

C1—C6	1.399 (3)	C10—C11	1.396 (2)
C1—C2	1.405 (2)	C10—H10	0.9500
C1—C7	1.468 (2)	C11—O3	1.359 (2)
C2—O1	1.377 (2)	C11—C12	1.394 (3)
C2—C3	1.387 (2)	C12—C13	1.389 (2)
C3—C4	1.385 (3)	C12—H12	0.9500
C3—H3	0.9500	C13—O4	1.376 (2)
C4—O2	1.373 (2)	C13—C14	1.387 (2)
C4—C5	1.391 (3)	C14—H14	0.9500
C5—C6	1.382 (3)	O1—H1	0.8400
C5—H5	0.9500	O2—H2	0.8400
C6—H6	0.9500	O3—H3A	0.8400
C7—C8	1.335 (3)	O4—H4	0.8400
C7—H7	0.9500	O5—H5A	0.890 (10)
C8—C9	1.469 (2)	O5—H5B	0.893 (10)
C8—H8	0.9500	O6—H6A	0.897 (10)
C9—C10	1.397 (2)	O6—H6B	0.894 (10)
C9—C14	1.402 (2)	O6—H6C	0.896 (10)
			
C6—C1—C2	116.74 (16)	C10—C9—C8	118.39 (16)
C6—C1—C7	123.22 (15)	C14—C9—C8	122.38 (15)
C2—C1—C7	119.97 (16)	C11—C10—C9	120.38 (16)
O1—C2—C3	120.45 (15)	C11—C10—H10	119.8
O1—C2—C1	117.62 (15)	C9—C10—H10	119.8
C3—C2—C1	121.93 (16)	O3—C11—C12	122.21 (15)
C4—C3—C2	119.39 (16)	O3—C11—C10	117.25 (16)
C4—C3—H3	120.3	C12—C11—C10	120.51 (16)
C2—C3—H3	120.3	C13—C12—C11	118.52 (16)
O2—C4—C3	119.43 (15)	C13—C12—H12	120.7
O2—C4—C5	120.23 (16)	C11—C12—H12	120.7
C3—C4—C5	120.32 (16)	O4—C13—C14	117.15 (15)
C6—C5—C4	119.44 (17)	O4—C13—C12	120.98 (15)
C6—C5—H5	120.3	C14—C13—C12	121.86 (16)
C4—C5—H5	120.3	C13—C14—C9	119.49 (15)
C5—C6—C1	122.11 (16)	C13—C14—H14	120.3
C5—C6—H6	118.9	C9—C14—H14	120.3
C1—C6—H6	118.9	C2—O1—H1	109.5
C8—C7—C1	126.20 (16)	C4—O2—H2	109.5
C8—C7—H7	116.9	C11—O3—H3A	109.5
C1—C7—H7	116.9	C13—O4—H4	109.5
C7—C8—C9	126.05 (17)	H5A—O5—H5B	104 (2)
C7—C8—H8	117.0	H6A—O6—H6B	119 (4)
C9—C8—H8	117.0	H6A—O6—H6C	109 (4)
C10—C9—C14	119.21 (15)	H6B—O6—H6C	106 (5)
			
C6—C1—C2—O1	−177.63 (15)	C1—C7—C8—C9	−176.59 (16)
C7—C1—C2—O1	5.2 (3)	C7—C8—C9—C10	173.61 (18)
C6—C1—C2—C3	1.8 (3)	C7—C8—C9—C14	−4.5 (3)
C7—C1—C2—C3	−175.39 (16)	C14—C9—C10—C11	0.3 (3)
O1—C2—C3—C4	−179.97 (16)	C8—C9—C10—C11	−177.84 (16)
C1—C2—C3—C4	0.7 (3)	C9—C10—C11—O3	179.27 (15)
C2—C3—C4—O2	175.96 (16)	C9—C10—C11—C12	0.9 (3)
C2—C3—C4—C5	−2.6 (3)	O3—C11—C12—C13	−179.80 (16)
O2—C4—C5—C6	−176.49 (17)	C10—C11—C12—C13	−1.5 (3)
C3—C4—C5—C6	2.1 (3)	C11—C12—C13—O4	−179.83 (16)
C4—C5—C6—C1	0.5 (3)	C11—C12—C13—C14	1.0 (3)
C2—C1—C6—C5	−2.3 (3)	O4—C13—C14—C9	−179.06 (15)
C7—C1—C6—C5	174.73 (18)	C12—C13—C14—C9	0.2 (3)
C6—C1—C7—C8	6.8 (3)	C10—C9—C14—C13	−0.8 (3)
C2—C1—C7—C8	−176.28 (18)	C8—C9—C14—C13	177.25 (16)

### Hydrogen-bond geometry (Å, º)

**Table:**

*D*—H···*A*	*D*—H	H···*A*	*D*···*A*	*D*—H···*A*
O1—H1···O4^i^	0.84	1.90	2.7329 (18)	173
O2—H2···O6^ii^	0.84	1.89	2.720 (2)	171
O3—H3*A*···O6^iii^	0.84	1.97	2.8045 (19)	169
O4—H4···O5^iv^	0.84	1.89	2.7259 (19)	170
O5—H5*A*···O1^i^	0.89 (2)	1.90 (2)	2.7879 (19)	172 (2)
O5—H5*B*···O2^v^	0.89 (2)	1.88 (2)	2.7449 (19)	161 (2)
O6—H6*B*···O2^ii^	0.90 (3)	1.95 (3)	2.720 (2)	143 (4)
O6—H6*C*···O6^vi^	0.90 (4)	1.87 (4)	2.7583 (19)	172 (5)

Symmetry codes: (i) −*x*, −*y*+1, −*z*+2; (ii) −*x*, −*y*+2, −*z*+1; (iii) −*x*+2, −*y*+1, −*z*+1; (iv) *x*+1, *y*, *z*; (v) *x*+1, *y*−1, *z*; (vi) −*x*+1, −*y*+1, −*z*+1.
